# Mechanical Dentistry

**Published:** 1878-08

**Authors:** C. S. Butler


### ARTICLE IV.

### *Mechanical Dentistry.*

BY C. S. BUTLER, D. D. S.

Read before the Eighth District Dental Society of the State of New York, held in Buffalo, April 30th, 1878.

In selecting subjects for discussion at this meeting, our members seem to have entirely overlooked the very important one of Mechanical Dentistry; and as it has fallen to the lot of your Committee to fill out the programme, I have selected this as a topic upon which to say a few words. Any by way of introduction, allow me to remark that I am thoroughly conscious of my inability to present you anything new on this subject, or advance ideas differing, probably, from those held by every member of this Society. But to my mind the subject is a very important one, and the main object of my bringing it before you will be found in the deep interest I feel in this direction, and the apparent neglect it is receiving at the hands of the profession.

During the few years I have had the honor of belonging to this profession, I have often been forcibly struck with the comparative ease and unconcern with which men divest themselves of this whole matter, devoting all their time and all their energies to the possibly more inviting field of *Operative Dentistry*. Indeed, to one unacquainted with the routine of a well regulated dental office, this whole subject of Mechanical Dentistry would seem to have no connection whatever with the dental profession, so few and careless are our considerations of it in our public meetings. There is a saying as true as it is old, that "from the abundance of the heart man speaketh;" and it does seem to me that if we come together here from year to year with the importance of this branch of our specialty resting upon our consciences, we will enter upon its discussion and go away greatly benefited for having exchanged our thoughts.

Understand me, gentlemen: I am not saying you do not give this matter due consideration in your offices. I am speaking simply of our meetings where every subject pertaining to dentistry ought to be fully and freely discussed; for it is only as we turn them over and over again, viewing them from every possible standpoint, that we may expect in any degree to attain the object of this organization, *i. e.*, the "dissemination of knowledge among its members." There is no subject too slight for our consideration, and no branch of our profession so unimportant as to delude us into the belief that there is no more to be learned on it.

I can never forget the impressions made upon my mind, while in college, by the very indifferent manner in which the students there, almost without exception, treated this subject of Mechanical Dentistry. I cannot now re-call one, out of the one hundred and fifty there present, who seemed to have any idea of this matter at all, or even a thought that it was related to the profession of dentistry. The idea of ever doing mechanical work had never entered the brain of many of them, and to be compelled to spend an hour or two in the laboratory each day was simply *awful*, while the requirements of the institution in this direction were almost enough to drive them from its lectures. Many of them attained high proficiency in Operative Dentistry; yet out of the forty-five graduates, I was told by the Professor, there was scarcely one able to pass a creditable examination in Mechanical Dentistry.

Now, gentlemen, what does this argue? Simply that there is neglect somewhere. It may not be in my office, it probably is not in yours; but it is somewhere, for those one hundred and fifty students all came from dental offices, and many of them from the best in the land. And what are we going to do about it? Shall we calmly fold our hands and sit idly by and see this branch of our chosen profession sink beneath the waves of indifference and charlatanism? I appeal to each of you, gentlemen, have we not a responsibility in this direction?

It requires no argument to convince every one of you that Mechanical Dentistry is a very important branch of our specialty. The term implies far more than one could expect to reach in a single paper. Its demands are on every hand, and its effects seen in nearly every household. It is a mistaken idea that the time is coming, as some men suppose, when there will be no demand for it. On the contrary. its demands are bound to increase; for notwithstanding all our boasted progress and comparative skill in saving the natural teeth, owing to the constantly degenerating condition of the human family, millions of these organs must be sacrificed every year, and their places supplied with artificial dentures. There is no dodging this point, and it behooves men to shape their studies accordingly. It is simply idle talk to say, as Dr. Smith, in the *Dental Register* for August, 1875, does say, that a young man without much literary preparation may enter the laboratory of some "surgeon dentist," and in six months' time acquire a sufficient knowledge of the business to pass himself acceptably with the people, fully meeting their expectations, or satisfying their limited ideas of what a mechanical dentist should be. The public demands more than this, and I for one will gladly welcome any measure which will take this matter from the hands of the irresponsible, and lodge it where it ought to be, with those accountable for what they do.

He who would most successfully practice this branch of our profession must be not only a good mechanic, but a good student as well. He should have a thorough knowledge of anatomy, surgery, physiology, physiognomy, chemistry, mineralogy, metallurgy and dynamics. These qualifications may be briefly stated as follows: *Surgery*, as it relates especially to all operations pertaining to the preparation of the mouth and the restoring the same to a healthy condition; *Mechanism*, or all that which pertains to the manual execution of the work, including impressions, models, dies, plates, mounting of the teeth, etc.; *General Chemistry*, or at least Special Chemistry, as it relates to the properties of the various substances used for artificial dentures, and the mode of preparing and componnding the different minerals, fluxes and oxides that are used in forming dental substitutes; *Metallurgy*, including the different processes of working, alloying, refining and adapting the various metals used in this branch of our profession; *Anatomy*,—and upon this head I would have the mechanical dentist the possessor of a thorough knowledge of the bony framework and the muscles, nerves, arteries and veins of the head and face, including the different locations, connections and functions of all the parts which give form and expression to the features of the face,—for upon this knowledge depends the restoration or distortion of the features of every person operated upon for an artificial denture; and lastly, that artistic qualification which combines all the preceding requirements, and constitutes the acme or crowning point of the whole.

It has been truly said that "the man with the best judgment is always the best dentist," which being interpreted is, the man with the best knowledge of not only the face and its surroundings, but of the whole human economy, is the best dentist, and this knowledge can be attained only by long and earnest study, together with a thorough and rigid discipline preparatory to entering upon the practice of his profession. True, there are those without this detailed knowledge of the human system, who after years of experience become expert and excellent mechanical dentists; but unfortunately, gentlemen, these are not they who labor in this direction, but prefer rather to intrust the whole matter to the judgment and skill of their inexperienced assistant. We hear a good deal in these days from members of our profession in regard to so much of this work going into the hands of quacks; and, as if to shift the responsibility for it, they say people have come to desire cheap work and low prices, and something must be done to protect ourselves against this perverted desire of our patients. Indeed, some have become so much alarmed for poor, deluded humanity (or themselves) that they would compel every man to become a graduate, or at least a licentiate, before he shall be privileged to practice dentistry at all; and to strengthen themselves in their position, hold up to our gaze the delightful effect legislation has had upon the profession in other States. Ohio is cited as a very paragon in this direction; and just as we are beginning to believe there is not a quack in the entire State, there comes to us in the *Dental Register* for April, 1878, the following advertisement:

SPECIAL NOTICE!

*Mechanical Work for the Dentists.*

We have made arrangements with mechanical dentists here to put up all styles of dental work for *our* customers at very reasonable prices. *The impression of the mouth or model from it, and the articulation, with sample of color and style of tooth, is all that need be sent.* Address———, Cincinnati, O.

What shall we say then? That there is nothing which savors of empiricism in an arrangement which not only permits but solicits my taking an impression, selecting a sample, and sending the same two or three hundred miles away, to be returned to me again in due time polished and ready for the mouth?

Well, gentlemen, this may be progress, but it is not dentistry; and I for one feel free to confess that I have very little sympathy in this whole movement for legislation in our State; for I hold this to be a free country, and am willing to grant unto every man the same rights and privileges I would require from him.

What we desire is not legislation, but education. Education in the profession and with the people; and not, I apprehend, until we have this, will an appreciable benefit be derived, even though we enforce all the laws in Christendom. What we want is, the best men in the profession to step forward and place this branch of our specialty on an equality with the operative; to lift it from the mire of indifference and carelessness which has so characterized it for the past few years, instructing their patients in the better methods, and demanding from them a remunerative fee. In this they hasten a re-action so long needed, and at the same time administer a rebuke to those who have merely "picked it up," and are loudest in mouthing their own achievements.—*Dental Advertiser.*

---

